# Antibiotic resistance: still a cause of concern?

**DOI:** 10.1097/OI9.0000000000000104

**Published:** 2021-06-15

**Authors:** Malcolm R. DeBaun, Cara Lai, Mark Sanchez, Michael J. Chen, L. Henry Goodnough, Amy Chang, Julius A. Bishop, Michael J. Gardner

**Affiliations:** a Department of Orthopaedic Surgery, Harborview Medical Center, Seattle, WA; b Department of Orthopaedic Surgery; c Stanford University School of Medicine; d Department of Medicine-Infectious Disease, Stanford University School of Medicine, Stanford, CA.

**Keywords:** antibiotics, bone infections, microbes, resistance

## Abstract

Antibiotic resistance remains a global public health concern with significant patient morbidity and tremendous associated health care costs. Drivers of antibiotic resistance are multifaceted and differ between developing and developed countries. Under evolutionary pressure, microbes acquire antibiotic tolerance through a variety of mechanisms at the cellular level. Patients after orthopaedic trauma are vulnerable to drug-resistant pathogens, particularly after open fractures. Traumatologists practicing appropriate antibiotic prophylaxis and treatment regimens mitigate infection and propagation of antibiotic resistance.

## 1 Introduction

Antibiotic resistance (ABR) is a major global health threat that has become ubiquitous across all health care settings, and continues to increase in scope and severity every year. In 2019, ABR was listed by the World Health Organization (WHO) as the fifth most significant threat to global health. The conservative estimate for annual deaths worldwide due to ABR is currently around 700,000.^[1]^ If left unchecked, ABR is projected to become one of the leading contributors to death with 10 million fatalities annually by 2050, outpacing other global threats such as cancer, diarrheal disease, diabetes, accidents, and violence.^[1]^ ABR also comes at a significant cost burden. A report released by the Organization for Economic Co-operation and Development (OECD) in 2018 predicted that in Europe, North America, and Australia alone, infections from drug-resistant organisms could cost up to USD 3.5 billion per year.^[2]^ Worldwide, drug-resistant infections could account for a loss of $100.2 trillion in GDP by 2050.^[1]^

Antibacterial resistance emerged as a concern in the 1970s, when the first drug-resistant strains of *Salmonella typhi* appeared.^[3]^ Resistance across a spectrum of microbes and their respective therapeutic agents has increased rapidly, coinciding with an overall increase in antibiotic usage worldwide. In the United States and Europe alone, 50,000 individuals die every year from Methicillin-resistant *Staphylococcus aureus* (MRSA) infection.^[1]^ Another study of nearly 700,000 drug-resistant infections in Europe demonstrated 39% of bacterial isolates were resistant to last-resort antibiotics.^[4]^ In Brazil, Indonesia, and Russia, 40% to 60% of infections are caused by drug-resistant microorganisms.^[5]^ Multidrug resistant gram-negative organisms are another emerging threat.^[6,7]^

Unfortunately, the development of new classes of antibiotics has lagged behind the emergence of resistance. Meanwhile, antibiotic access and utilization have increased. While high-income countries have maintained a steady rate of consumption, lower and middle-income countries (LMICs) are increasing antibiotic utilization. This is influenced by lack of surveillance, low barriers for use and associated misuse, decreased costs, and high availability.^[8]^

## 2 Drivers of antibiotic resistance

Microbes inherently possess genetic variability, with mechanisms that confer resistance to different classes of antibiotics. The 3 main contributing mechanisms include intrinsic resistance, genetic mutations, and horizontal gene transfer (including plasmid-mediated transfer). ^[9]^ Additionally, enzymatic deactivation of antibiotics, cell wall modifications, efflux pumps removing toxic products, and modified drug-target sites contribute to ABR on a cellular level.^[10]^ These inherent mechanisms driving microbial evolution inevitably overcome antibiotics over time by selecting for resistant strains. Indiscriminate utilization of antibiotics only accelerates ABR.

**Figure 1:**
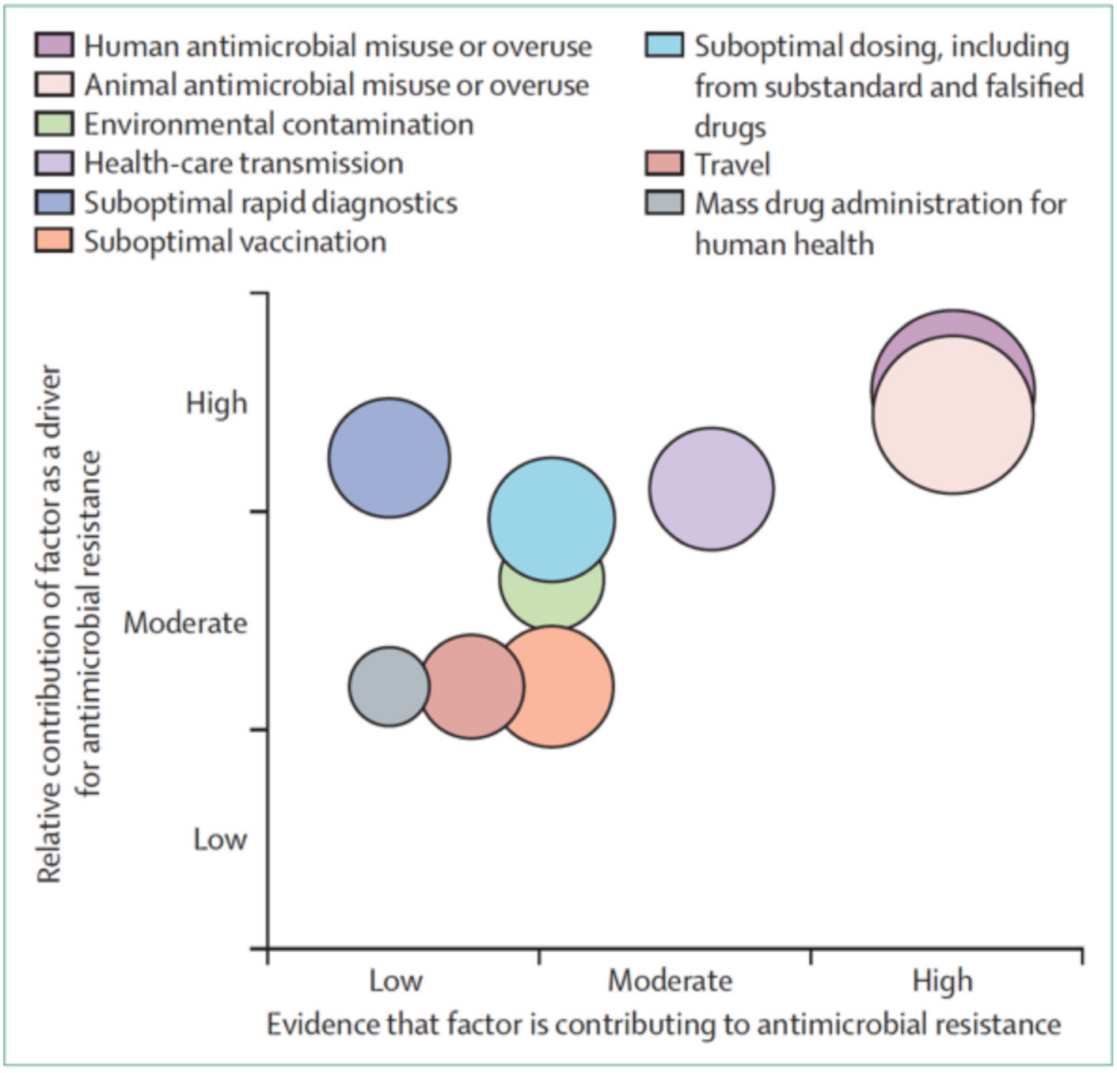
Societal drivers of antibiotic resistance displayed by evidence and contribution. Reprinted from *The Lancet*, Vol. 387, Holmes AH, Moore LSP, Sundsfjord A, et al., Understanding the mechanisms and drivers of antimicrobial resistance, pages 176–187, Copyright (2016), with permission from Elsevier.

Societal influences contribute to ABR in high-income countries and LMICs (Fig. 1). Noncompliance with appropriate use criteria for antibiotic administration due to miseducation can be problematic in both settings, especially when prescribed for nonbacterial infections or noninfectious pathologies.^[11,12,13,14,15,16]^ Notably, practitioners in private practice tend to overprescribe antibiotics compared with those working in community hospitals.^[17,18]^ Nosocomial infections represent a large number of drug-resistant infections, with transmission between patients and healthcare workers, who may practice infrequent handwashing and sanitation procedures. The routine prophylactic administration of antibiotics for invasive medical or surgical procedures can also contribute to ABR.^[19]^

Resistance rates in LMICs rise at a 4–7× faster rate compared with high-income countries, secondary to increases in antibiotic utilization.^[5]^ In LMICs, available antibiotics may be expired or substandard.^[11,20,21]^ Along with the disproportionate increase in ABR prevalence, LMICs can lack the infrastructure necessary to effectively track multidrug-resistant infections or enforce protocolized antibiotic administration. A report from the WHO in 2014 found great variation in level and quality of antibiotic surveillance programs in LMICs, and subsequently founded the Global Antimicrobial Resistance Surveillance System to address this critical gap.

There are some unique drivers of ABR in developed countries. The proliferation of widespread antibiotic use with minimal regulation in the agricultural industry is a huge contributor to AMR.^[22]^ Antibiotics are given to livestock in high-income countries not only for prophylaxis but also to stimulate animal growth.^[23,24]^ Antibiotic-resistant bacteria of animal origin are then transmitted to human hosts through meat consumption or contaminated water supply.^[25]^ Also, international travel can lead to the dispersal of drug-resistant organisms between continents.^[1]^

## 3 Antibiotic resistance in fracture surgery

Drug-resistant organisms are particularly prevalent in deep infections after fracture fixation. A study of 214 postoperative infections associated with fracture surgery demonstrated that 32% of all infections had *MRSA* present.^[26]^ Another 32% of infections speciated gram-negative rods (GNR), which were more prevalent after surgeries involving the pelvis and proximal femur. A total of 4% of these GNR were multidrug resistant. There were 14% of patients who grew enterococcus, of whom 20% were resistant to vancomycin. Overall, 36% of patients grew at least 1 antibiotic resistant organism.

Open fractures are associated with increased risk of infection. In a Brazilian study of 11,030 patients who underwent internal fixation for open (17.1%) and closed fractures, the overall incidence of infection was 6%, which disproportionally occurred more in open fractures (14.7%).^[27]^ The overall incidence of MRSA was 72%. A total of 14% of patients with open fractures grew enterococcus isolates, which were resistant to vancomycin 24% of the time. *Acinetobacter baumannii* and *Pseudomonas aeruginosa* were the most common GNRs isolated in open and closed fractures, respectively, both of which exhibited low rates of susceptibility to carbapenems. A retrospective cohort study of 202 various open fractures demonstrated a 10% incidence of infection. A total of 25% of these infections were MRSA. The overall incidence of MRSA infection after open fractures was 2.5% in their population. Amongst those patients with infection, 55% grew at least 1 GNR.^[28]^ Another study of open fractures showed a 56% incidence of MRSA infection amongst those that were culture positive.^[29]^

Drug-resistant infections have been extensively profiled in patients with hip fractures. In a study of 3686 patients at a British teaching hospital, 48% of patients with infection were attributed to MRSA (39 total patients). An MRSA infection after hip fracture was associated with increased costs, length of stay, and mortality compared with non-MRSA infection. In a multicenter study of 22 hospitals in France, the most common infectious isolate after femoral neck fracture was MRSA (32%).^[30]^ Five percent of patients with a hip fracture in the United Kingdom were colonized with MRSA, as determined by nasal swab on admission.^[31]^ A high proportion of these patients had been hospitalized within the previous year (81%).

Another study looked into variations in bacterial pathogens causing infection after fracture treatment.^[32]^ They compared the proportion of causative isolates from one 5-year historical period to the subsequent 5-year study period. During this time, there was a reduction in the proportion of MRSA in the overall sample from 32% to 18%. They proposed several reasons to account for the decline in MRSA based upon changes to their clinical practice. First, they cited the increased prevalence of topical vancomycin powder at closure could have accounted for the decreased infection rate. They also suggested that routine preoperative skin preparation with chlorhexidine might have resulted in decreased proportions of MRSA. Additionally, they emphasized national declining rates of MRSA possibly related to infection control measures such as chlorhexidine baths, nasal decolonization, surveillance techniques, aggressive device removal, and early isolation of MRSA-positive patients or therapeutic measures such as topical vancomycin application and antibiotic coating of various devices.^[33]^

## 4 Antimicrobial stewardship in fracture surgery

Although routine antibiotic prophylaxis to prevent infection after fracture surgery has been widely accepted as standard of care for decades, some questions remain as to specific regimens in the era of evolving antibiotic resistance.^[34,35]^ In a recent survey of OTA members, 96% of surgeons recommended cefazolin as first-line prophylaxis in closed fractures. Most surgeons (58%) administered antibiotics within 1 hour of incision with redosing for 24 hours postoperatively, whereas 39% used a single dose and the remainder varied depending upon the clinical scenario.^[36]^ Practice guidelines from multiple societies endorse routine antibiotic administration prior to incision with redosing to maintain therapeutic systemic levels up to 24 hours after skin closure.^[37,38]^ There are a few meta-analyses that suggest single dosing has similar outcomes as multiple doses, but these conclusions are based upon low-quality data.^[39,40,41,42]^ Therefore, specific recommendations as to single versus 24-hour dosing for closed fractures remain unclear based upon existing evidence.

Intravenous antibiotic administration is critical in preventing infection in open fractures, but the optimal duration remains controversial. Currently, coverage for gram-positive organisms is recommended with the addition of gram-negative coverage for type III open fractures and anaerobic coverage against *Clostridium* species for those wounds with fecal or soil contamination.^[43]^ Systemic antibiotics administered within 66 minutes of open injury are protective against deep infection.^[44]^ Importantly, timing of surgical debridement appears not to influence infection risk if performed within 24 hours of injury.^[45]^ Although duration of antibiotics is still debated, high-quality evidence suggests intravenous antibiotic administration no longer than 24 hours after wound closure optimally prevents postoperative infection after open fracture. Routine administration of antibiotics beyond this point is unnecessary in most clinical scenarios and potentially contributes to the development of antibiotic resistance. A summary of our institutional recommendations for prophylactic antibiotic administration is listed in Table 1.

**Table 1:** Summary of prophylactic intravenous antibiotics for fracture surgery

Fracture type	Antibiotic target (e.g.)	Duration
Closed	Gram-positive coverage (Cefazolin)	Up to 24 h postoperative
Open		
Type I	Gram-positive coverage (Cefazolin)	Up to 24 h postoperative
Type II	Gram-positive coverage (Cefazolin)	Up to 24 h postoperative
Type III	Gram-positive and gram-negative ± anaerobic coverage if contaminated with feces/soil (Ceftriaxone ± one dose of Penicillin G)	Up to 24 h after wound closure

Improved antibiotic stewardship does not translate to increased infection risk. A level-one academic trauma center implemented a protocol to guide antibiotic prophylaxis in open fractures based upon Gustilo-Anderson type.^[46]^ Type I/II fractures were administered cefazolin (clindamycin if allergy) and ceftriaxone (clindamycin and aztreonam if allergy) was administered for Type III fractures. Compared with pre-protocol controls, there was no increase in infection rates despite decreased utilization of aminoglycoside and glycopeptide (vancomycin) antibiotics. Although the reduction in broad-spectrum antibiotics may theoretically mitigate selection pressure for ABR, there was no difference in the emergence of multidrug resistance organisms or MRSA between pre- and postprotocol implementation in this study.

Appropriate indications for local delivery of antibiotics are evolving. A meta-analysis of open fractures reported a risk reduction of 11.9% in fracture-related infection if additional local antibiotics were given prophylactically.^[47]^ However, there was considerable heterogeneity, bias, and low-quality data that confounded translation of these results into clinical practice. A study group recently presented results of a large, randomized, multicenter trial in which vancomycin locally delivered in patients with open fractures before wound closure was compared to patients who did not have adjunctive local antibiotic delivery.^[48]^ The rate of infection in the topical vancomycin group was lower with a relative risk reduction of 0.66 (95% CI 0.42–1.02; *P* = 0.07). This reduced infection risk was attributed to effective prophylaxis against gram-positive organisms, as the rate of gram-negative infections was unchanged. The long-term ramifications of routine adjunctive local delivery of antibiotics in open fractures with respect to ABR remain unclear.

Novel strategies to improve antibiotic stewardship are being developed.^[43,49]^ Advanced diagnostic techniques, such as bacterial or metagenomic sequencing, allow for efficient identification of causative organisms and targeted antibiotic administration. Also, bacteriophages and other nonantibiotic therapies the activate the host's innate immune system may translate into mainstream treatments but are currently developmental. The ideal duration of antibiotics for both prophylaxis and proven infection is still unclear and also warrants future investigation.

## 5 Conclusion

Antibiotic resistance remains a significant global health issue. Bacteria inherently evolve resistance to antimicrobials over time which is accelerated through selective pressures generated by antibiotic exposure. Orthopaedic trauma surgeons should be aware of evidence-based prophylaxis and treatment strategies to effectively prevent and manage postoperative infection without contributing to the development of antibiotic resistance through indiscriminate prescribing.
